# Ensembl variation resources

**DOI:** 10.1093/database/bay119

**Published:** 2018-11-06

**Authors:** Sarah E Hunt, William McLaren, Laurent Gil, Anja Thormann, Helen Schuilenburg, Dan Sheppard, Andrew Parton, Irina M Armean, Stephen J Trevanion, Paul Flicek, Fiona Cunningham

**Affiliations:** European Molecular Biology Laboratory, European Bioinformatics Institute, Wellcome Genome Campus, Hinxton, Cambridge, UK

## Abstract

The major goal of sequencing humans and many other species is to understand the link between genomic variation, phenotype and disease. There are numerous valuable and well-established variation resources, but collating and making sense of non-homogeneous, often large-scale data sets from disparate sources remains a challenge. Without a systematic catalogue of these data and appropriate query and annotation tools, understanding the genome sequence of an individual and assessing their disease risk is impossible. In Ensembl, we substantially solve this problem: we develop methods to facilitate data integration and broad access; aggregate information in a consistent manner and make it available a variety of standard formats, both visually and programmatically; build analysis pipelines to compare variants to comprehensive genomic annotation sets; and make all tools and data publicly available.

## Introduction

One of the most important challenges in contemporary biomedical research is to understand how changes in genome sequences lead to different phenotypes and influence disease. Collecting and interpreting genomic sequence variation is the key to addressing these and other questions. Indeed, recent results have provided insight into disease susceptibility (1), treatment response (2), development of desirable traits in animals (3) and crop plants (4) and understanding population genetics in many species (5). Human genetics study designs in particular are transitioning from targeting a handful of candidate genes to full exome or genome sequencing to find trait-associated loci in individuals, families or populations (6, 7). In all study designs, sites of genome variation alone are insufficient to draw conclusions. To interpret results, it is essential to have access to additional data such as allele frequencies in reference populations, reported disease associations and predicted functional impact on genes (8). To facilitate this important and growing area of research, Ensembl identifies, integrates and organizes comparable data from different sources into a system that can be queried and browsed using uniform access and analysis methods.

Here, we describe how the variation and phenotype data sources incorporated into Ensembl are harmonized, visualized and programmatically accessed. We focus on data sources relevant for genome interpretation, including for understanding human phenotypes and disease. Major changes since our last report [9, that described the variation resources present in Ensembl release 56 (September 2009)] include the incorporation of more extensive phenotype and disease annotation in multiple species, the inclusion of variation citation data, improved allele normalisation and equivalence algorithms and infrastructure changes to support the presentation and visualisation of many millions of genotypes.

**Figure 1:**
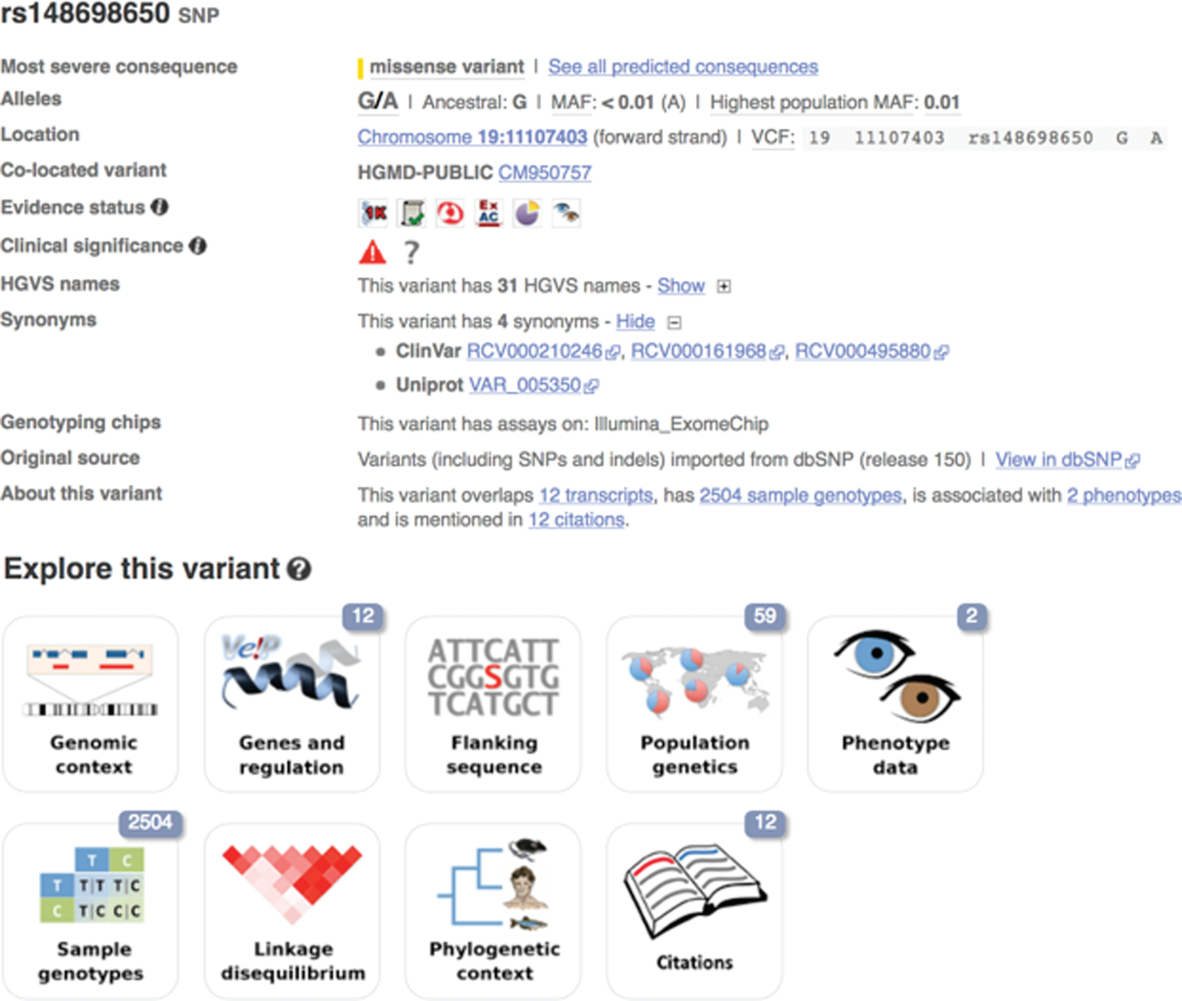
A variant summary view for rs148698650 that displays the global minor allele frequency from the 1000 Genomes Project, other variants at the same location and links to projects providing additional information about the variant. URL: https://www.ensembl.org/Homo_sapiens/Variation/Explore?v=rs148698650

## Data access

### The genome browser

For analysis of small areas of the genome, such as variation in a single gene or transcript, visual displays remain the key to explore, analyse and communicate scientific findings. We provide access to the data we incorporate through several different interactive displays via the Ensembl genome browser (www.ensembl.org).

From the Ensembl home page, searching for genomic regions, genes, phenotypes, diseases or variant identifiers provides access to different data views. For example, variant-specific web pages (see Figure 1) are a convenient way to visualize data aggregated from a wide range of different variant-focused projects combined with the results of our analyses. The information is presented both as a summary and as a set of detailed graphical views and tables. These are described in more detail below.

**Figure 2:**
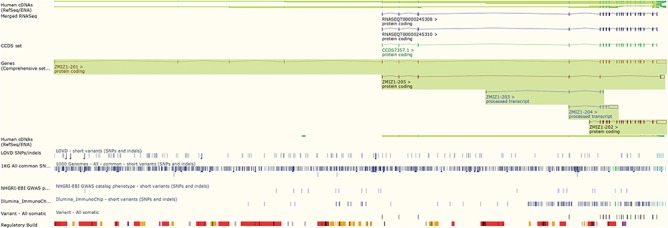
The ‘Region in detail’ view showing 5 of the available 66 tracks of variant data. URL: https://www.ensembl.org/Homo_sapiens/Location/View?r=10:79069035-79316528

The Ensembl genome browser also provides displays of genomic regions in which variants are presented alongside transcripts, regulatory features and conservation information. Variants are coloured by the most severe predicted impact they have on gene function. This ‘Region in detail’ view (see Figure 2) can be configured to display tracks of specific variants, such as those from ClinVar, those with phenotype annotations or those assayed on selected genotyping arrays, such as the Illumina ImmunoChip (10) and the Affymetrix Chicken600K array (11).

Our dedicated gene pages include tables describing all variants in the gene region with many filtering options including by consequence or variant type. These are configurable to improve readability and analysis capabilities, especially in genes with a large quantity of variant data. We also provide configurable phenotype pages that show variants and genes associated with a phenotype, trait or disease. On both pages, data are imported from multiple sources and presented in a single table for easy comparison.

### Application programming interfaces

To facilitate direct data access for flexible analysis and to support the web displays described above, we have developed and extended our methods for rapid bulk data retrieval. Custom queries can be written using our mature and comprehensive Perl application programming interface (API), for which extensive documentation and detailed tutorials are available on the Ensembl website. We have also implemented a Representational State Transfer (REST) API (12) for language agnostic data access. Together, these enable convenient integration of our tools and data into multiple other websites and analysis pipelines.

### Variant Effect Predictor

The data assembled in the Ensembl databases can be used to annotate variants and predict their functional impact using the Ensembl Variant Effect Predictor (VEP) tool (13). VEP provides a simple, yet powerful, interface using the Ensembl API, which enables the user to annotate an entire human genome (around 4 million variants) in less than an hour and an exome (around 200 000 variants) in less than 5 minutes. It can be accessed via a web interface, a stand-alone script and a REST API. The web interface is integrated with other Ensembl views allowing navigation directly from VEP results to detailed information on any transcripts or to previously known variants that match an input variant. The stand-alone script is highly configurable and can be extended to incorporate additional data from Ensembl (using the Perl API) or other sources. For more detailed information, see (13).

## Heterogeneous data integration

Over the past 8 years, we have increased the number of supported species, the range of data types we hold and volume of data we store, analyse and serve. For example, we have extended our schema to include variant data citation data and greatly improved our management of phenotype and disease annotations to incorporate additional data sources.

### Short variants

The Ensembl variation databases currently contain publicly available data for 20 vertebrate species from sources including dbSNP (14), the European Variation Archive (EVA) (https://www.ebi.ac.uk/eva/) and ClinVar (15). To allow all information for a locus to be considered together, we collate data where possible and index the merged records with identifiers from multiple resources. This enables searching directly with accession numbers from multiple databases including dbSNP, UniProt (16), PharmGKB (17) and ClinVar.

Since our last report, the number of short variants held in Ensembl databases has risen from 56 million to over 1166 million as of Ensembl release 93 (July 2018). The distribution of data across species has also changed due to major variant discovery efforts in human and livestock species. Genotype and allele frequency data are available for an increasing number of species, from sources such as the Mouse Genomes Project (18) and the sheep and goat focused NextGen project (19).

**Figure 3:**
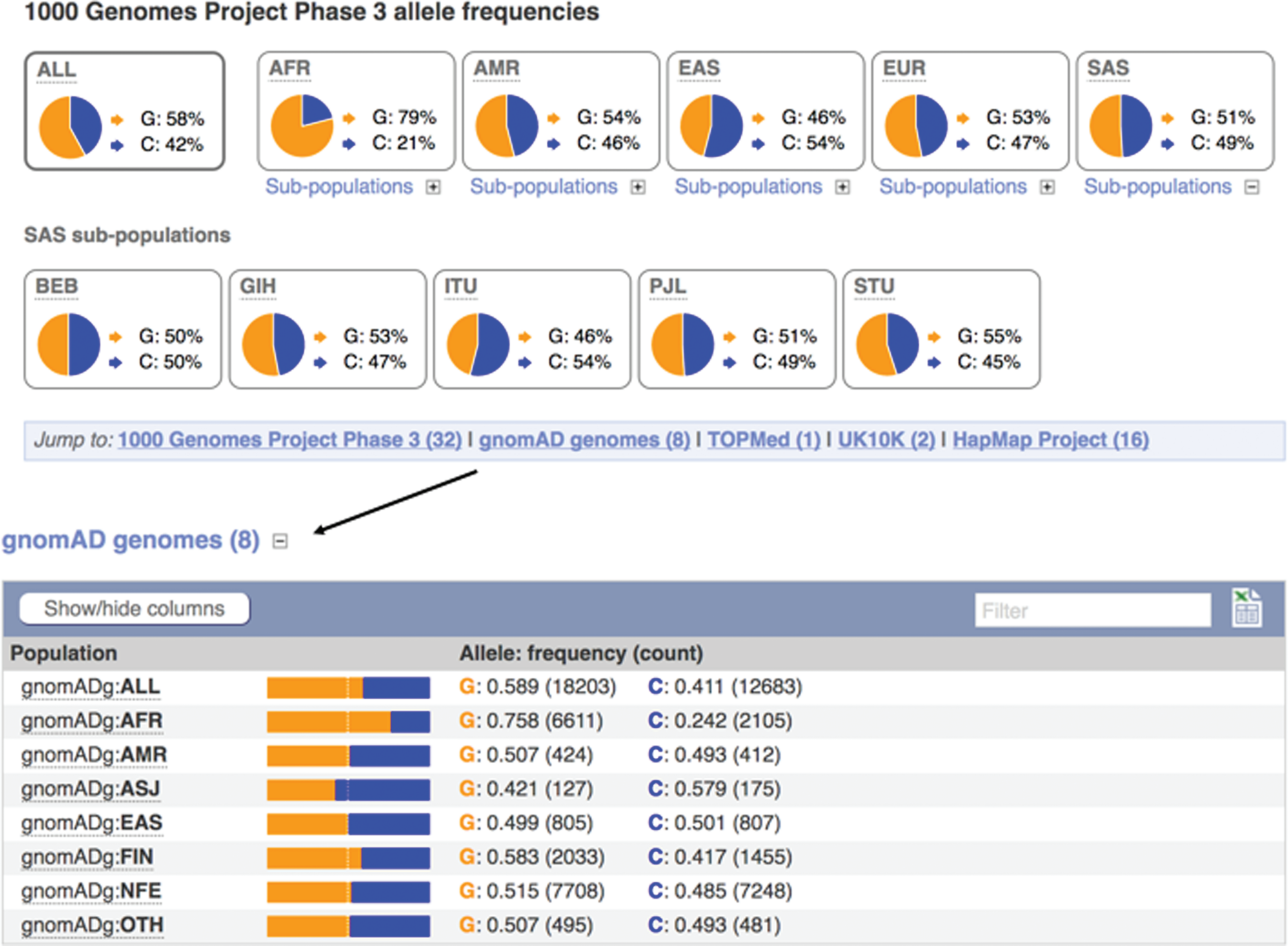
Displays of the frequency spectrum of variant rs1333049 in the 1000 Genomes Project and Genome Aggregation Database panels. URL: https://www.ensembl.org/Homo_sapiens/Variation/Population?v=rs1333049

The vast majority of our variant data are imported from (*The National Center for Biotechnology Information*) NCBI’s dbSNP database. As an archive, dbSNP accepts all submitted variants, so entries vary in level of supporting evidence. To identify the most robust data from these multiple submissions, we have extended our quality control process to have two stages. As previously described, variants are considered as ‘suspect’ if they show certain characteristics, such as a mismatch between the reported variant alleles and the reference genome sequence at the specified location. We now also summarize the evidence supporting each variant such as inclusion in a large-scale reference project. Details of our data and processing procedure are listed at: https://www.ensembl.org/info/genome/variation/index.html.

When seeking to identify somatic mutations or variants involved in rare disease, it is standard practise to filter the variants found in a clinical sample for those already known to be common in the population (8). We therefore provide frequency data from a number of reference sets including the 1000 Genomes Project (20), which sequenced, at low coverage, the full genomes of 2504 individuals from 26 populations and the Genome Aggregation Database (gnomAD) (21), which collected and analysed the exomes of 123 136 individuals, and the full genomes of 15 496 individuals from 7 populations. Our variant ‘Population Genetics’ page displays frequency distributions across the typed populations (see Figure 3). To show how common an allele is in any assayed ethnic group, we report the highest minor allele frequency observed in any sample set, including 1000 Genomes Project’s regional sub-populations. This facilitates improved filtering of common variants from potential disease alleles.

### Structural variants

**Figure 4:**
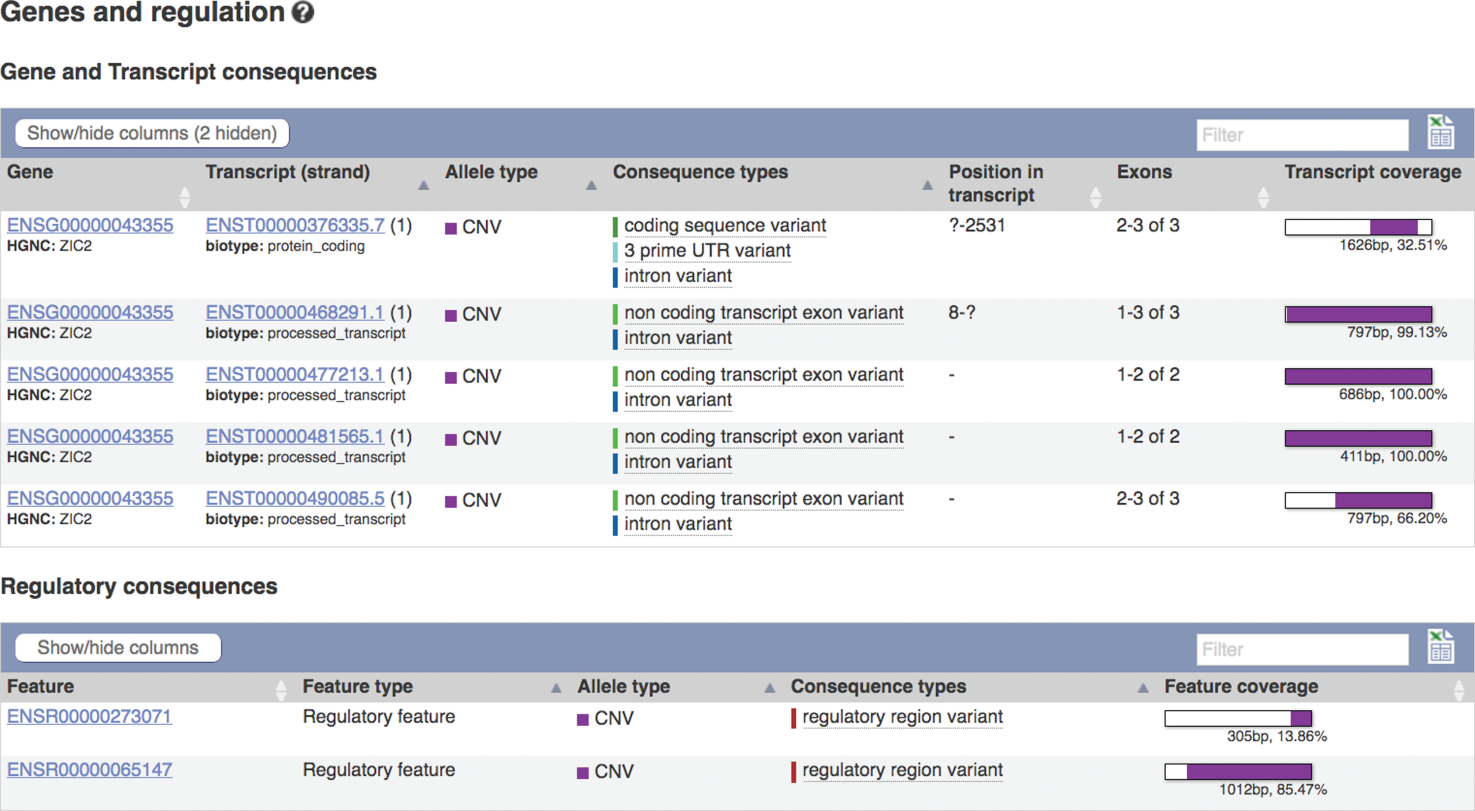
Transcripts and regulatory features overlapping SV nsv916030. URL: https://www.ensembl.org/Homo_sapiens/StructuralVariation/Mappings?sv=nsv916030

The European Molecular Biology Laboratory European Bioinformatics Institute (EMBL-EBI) Database of Genomic Variants archive (DGVa) and NCBI’s dbVar (22) are peer archives of structural variant (SV) information. We import SV data from these archives for nine species. To facilitate filtering, we annotate SVs with any transcripts or regulatory features they overlap (see Figure 4) and report variant type and consequence using standardized Sequence Ontology (SO) terms (23). We also calculate population frequencies for SVs discovered in the 1000 Genomes Project.

### Citations

Publications contain valuable information including variant to disease associations, but it can be cumbersome to collate an extensive list of references. Variant identifiers are manually extracted from the literature by projects such as the The National Human Genome Research Institute -European Bioinformatics Institute genome wide association study catalog (NHGRI-EBI GWAS Catalog) (24); computationally mined by the University of California, Santa Cruz (25) and by Europe PubMed Central (26); and referenced in submissions to dbSNP and ClinVar. These alternative approaches yield different sets of data. Since our last report, we have implemented data collation and access methods for citations from all of these sources, and citations are now available in Ensembl for nine species. Our variant ‘Citations’ page lists publications that describe the variant, with links to abstracts and full text where available. We thereby avoid the need to collate lists of references by providing immediate access to a simple overview of the publications discussing a particular variant and easy navigation to detailed reports.

### Phenotype and disease information

We aggregate multiple distinct sources of phenotype, disease and trait annotations into a common structure to mitigate many of the challenges of using these data. At present, there is no common format used to exchange such data and projects often use different conventions to describe the same condition. The type of information available for these annotations is also highly heterogeneous and dependent on data source. Annotations can be associated with variants, SVs, genes or quantitative trait loci (QTL), causing additional complexity when seeking all phenotype information for a genomic locus. Our data integration methodology facilitates the analysis of heterogeneous annotations from different sources via a single interface.

For human, our primary data sources include ClinVar, OMIM (27) and the NHGRI-EBI GWAS Catalog. For other species, we import data from the Animal QTL database (28), Online Mendelian Inheritance in Animals (OMIA) (29) and a number of species-specific projects such as the Rat Genome Database (30), the Zebrafish Model Organism Database (31) and the International Mouse Phenotyping Consortium (32). We hold phenotype descriptions as used by the data providers and, to facilitate improved querying across conditions described differently in different studies, we map these to ontology terms in the experimental factors (33), human phenotype (34), clinical measurement (35) and mammalian phenotype (36) ontologies, where possible. In Ensembl release 93 (July 2018), 80% of the phenotype descriptions present in our human database were mapped to an ontology term. The mean number of descriptions mapping to an ontology accession is 2.2 but over 8% of accessions map to 5 or more descriptions. An extreme example is deafness (EFO:0001063), which is linked to 125 different descriptions, including ‘Deafness, autosomal recessive, 53’ and ‘Deafness, autosomal dominant, 20’.

**Figure 5:**
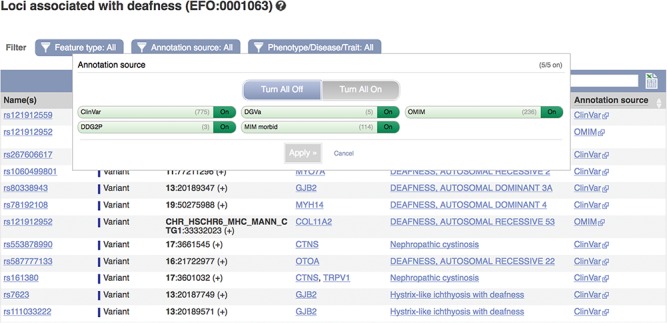
A phenotype table showing variants associated with deafness. URL: https://www.ensembl.org/Homo_sapiens/Phenotype/Locations?oa=EFO:0001063

These data can be accessed via the Ensembl genome browser by searching for disease or phenotype descriptions or ontology terms. Our phenotype pages display a table of genes, variants and QTLs annotated as being associated with the phenotype, trait or disease (see Figure 5). By default, locus names, genomic locations and the source of the annotation are displayed with links out to the data source and any publications in PubMed. These links provide access to more detailed evidence supporting the assertions. We also display clinical significance assertions and review status from ClinVar, where available, using clear icons to distinguish the different statuses and reporting when conflicting reports have been submitted. When annotations are viewed clustered by ontology term, similar diseases or those sharing phenotypes are displayed. Our variant ‘Phenotypes’ page lists any phenotypes, traits or diseases that are reported to be associated with the variant. Our gene ‘Phenotypes’ page shows annotations grouped by gene and also displays phenotypes associated with orthologues in other species, as it is possible that function is shared. Our APIs also provide data access by variant, gene, region and phenotype ontology term.

### Data sets with licensing or access restrictions

Some valuable data have license restrictions that prohibit redistribution or limit the detail that can be shown in Ensembl. In other cases, distribution is restricted by specific consent agreements with the research participants. In both cases, we seek to maximize data discoverability while complying with known restrictions. For example, we incorporate the public versions of the Human Gene Mutation Database (HGMD) (37) and the Catalogue of Somatic Mutations in Cancer (COSMIC) (38) data sets into Ensembl. We report these data in region-based and identifier-based searches and provide links to each project’s website for additional information. As of Ensembl release 93 (July 2018), there are over 139 000 HGMD identifiers and over 4 million COSMIC identifiers with genomic locations. Variant locations from the DECIPHER (39) project and Leiden Open Variation Database (40), which are restricted by consent agreements, are not available via our APIs but can be viewed as tracks on the ‘Region in detail’ view with links to the project websites.

A full list of data sources and versions used is available at: https://www.ensembl.org/info/genome/variation/species/sources_documentation.html.

## Variant annotation

To enable integration and discovery of the increasing wealth of variant data in many species we have implemented a number of high-throughput annotation pipelines since our last report. We have also developed tools and REST services to provide dynamic data analysis.

**Figure 6:**
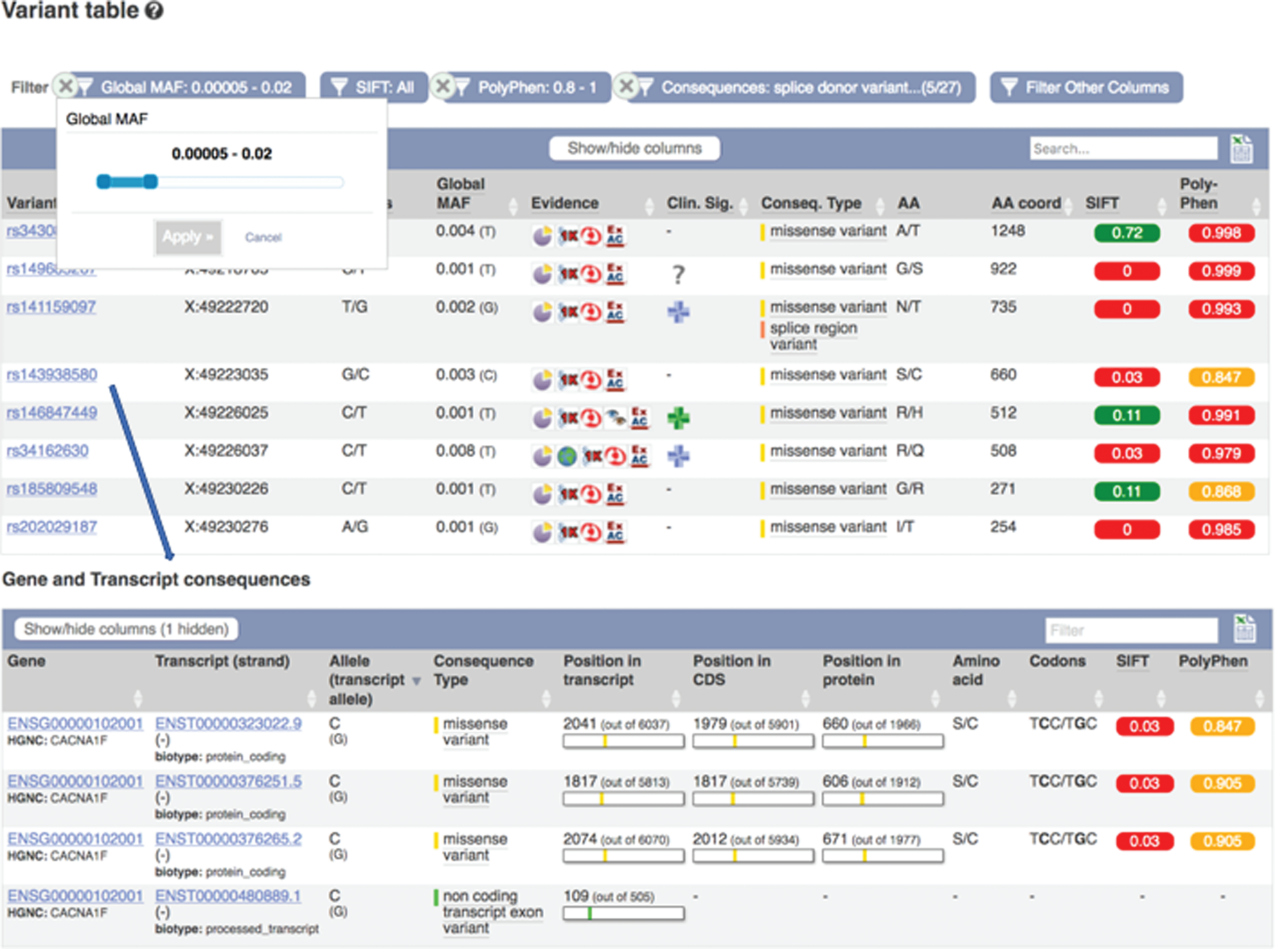
Part of a table showing predicted consequences for the variants overlapping transcript ENST00000323022.9. Table of transcripts overlapping variant rs143938580. URLs: https://www.ensembl.org/Homo_sapiens/Transcript/Variation_Transcript/Table?t=ENST00000323022, https://www.ensembl.org/Homo_sapiens/Variation/Mappings?v=rs143938580

### Predicted variant effect on gene function

Ensembl creates comprehensive gene annotation for over 100 vertebrate species (41) and regulatory element annotation for human and mouse (42). We analyse variants in Ensembl with respect to this annotation and predict the consequences of each allelic change on any overlapping feature to provide guidance as to its possible functional impact. We use SO terms to describe these consequences, enabling comparison with other annotation sources and querying by both specific and generic terms (for example, both missense and synonymous variants can be extracted using the generic term ‘exonic’). To assess the potential deleteriousness of missense variants, we employ the Sorting Intolerant From Tolerant (SIFT) (43) and PolyPhen2 (44) packages, which use protein homology and structural information to predict the impact of a change in amino acid sequence on protein stability and function. SIFT results are available for our most highly accessed species—including human, chicken, cow, dog, goat, horse, mouse, pig, rat and zebrafish—while PolyPhen2 is specific to human. Many additional functional effect algorithms are available for human variants via VEP.

These results can be viewed grouped by gene or variant. Our variant ‘Genes and regulation’ page displays the predicted functional consequences for each transcript and regulatory feature the variant overlaps; the position in the transcript, CDS and protein sequences with amino acid and codon change are listed where available. Our transcript ‘Variant’ page (see Figure 6) displays the predicted functional consequence of each variant in the region, with amino acid position and change, and SIFT and PolyPhen2 scores, where available. To help assess potential deleteriousness, the evidence supporting each variant and the minor allele frequency in the 1000 Genomes Project samples is reported. These results are presented in a table that can be interactively filtered on any of these attributes as well as variant type, location and data source.

### Conservation

Allele conservation is one of the strongest predictors that genomic sequence modifications are not tolerated (45). To indicate allele conservation, we use Ensembl’s comparative genomics resources (46) to display the sequence flanking a variant aligned with genomic sequence from relevant sets of other species on the variant ‘Phylogenetic context’ page. We also predict the ancestral allele at each variant location in primate species.

**Figure 7:**
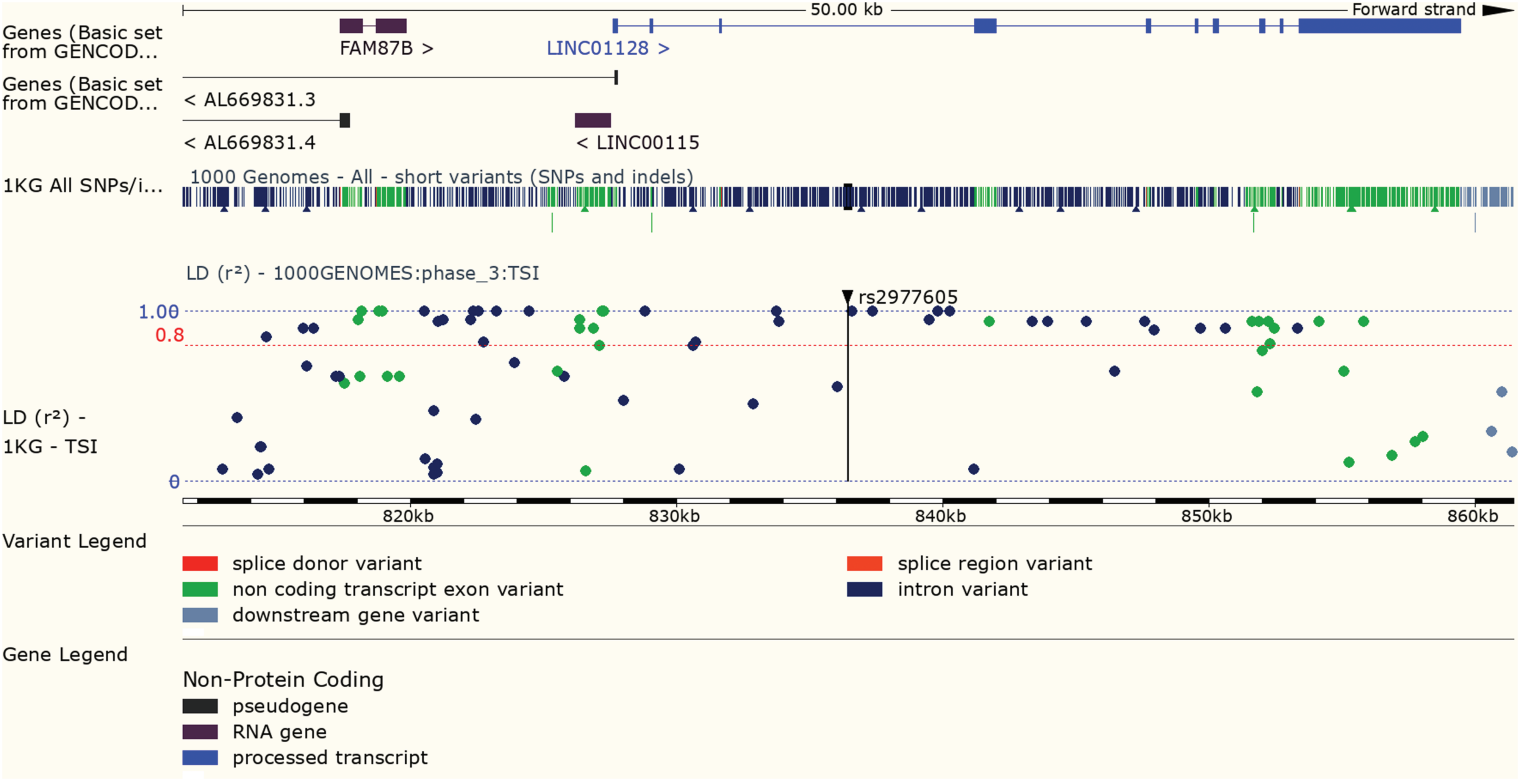
LD results for a variant can be viewed adjacent to gene structure in both Manhattan and Haploview style plots. URL: https://www.ensembl.org/Homo_sapiens/Variation/LDPlot? v=rs2977605;pop1=373537

### Allele equivalence

Repositories suffer from the lack of a consistent variant reporting standard, allowing equivalent insertions or deletions in repetitive sequence to be reported at different locations depending on whether the change is left or right justified (47). Problems in interpretation can arise when disease annotations are attached to a variant at one location and frequency information to an equivalent variant at a different location. We have implemented a method to systematically extract all variants in overlapping five megabase regions, normalize each allele individually to the most 3′ position at which it can be described and identify variants with equivalent alleles. We list variants with equivalent alleles on our variant page to allow scattered information about a genomic change to be considered together. Over 3 million human variants have equivalent alleles listed in our current release.

### Linkage disequilibrium

Variants found to have associations with disease in large-scale studies are rarely causative, so an understanding of linkage disequilibrium (LD) in the region around the variant is essential. Our variant ‘Linkage Disequilibrium’ page displays LD results calculated in the sample populations typed in the 1000 Genomes Project, supporting tagSNP selection and the interpretation of association results (see Figure 7). In March 2016 we added a REST service, which can be used in analysis pipelines to access both D’ and r^2^ values by region or variant, and in December 2017, we released a web tool that provides LD results for a single variant, group of variants or region.

### Transcript haplotype frequencies

The reference human genome is a mix of sequences from several different individuals (48) and, as such may contain regions, including very rare alleles, which have not been observed together in a single individual. In studies investigating disease or drug response, it is useful to know the protein forms common in different populations, rather than considering only the translation represented by the reference sequence. (58).

In August 2016, we first provided protein haplotype sequence frequency data for human genes, calculated using the phased genotypes from the 1000 Genomes Phase 3. We consider the effect of all variant alleles acting together on protein function and include SIFT and PolyPhen2 predictions of deleteriousness. Each haplotype is assigned a description showing the allele change and the frequency in the continental populations is calculated. These data are available in both the genome browser (from the ‘Haplotypes’ tab on the human Transcript pages) and via dedicated REST endpoints. A small number of reference haplotypes were unobserved in the 1000 Genomes samples. The Genome Reference Consortium is now reviewing these.

### Variant Recoder

Variants can be named in a variety of ways in literature and database resources, (for example, the identifiers ‘ENSP00000420705.2:p.Ser737Asn’, ‘NM_007294.3:c.5522G>A’, ‘rs80357368’ and ‘RCV000236784’ all refer to the same variant) causing difficulties in interpretation. To resolve this issue, we have implemented a REST service to return Human Genome Variation Society (HGVS) descriptions and other known names for an input identifier. The Variant Recoder takes accessions from databases such as dbSNP, UniProt and ClinVar as input as well as HGVS at genomic, transcript and protein level. It also decodes some common forms of incorrect HGVS descriptions, such as gene name with a protein level change, where possible. Warnings are issued when invalid HGVS is input, or results are potentially ambiguous.

## Methods

Ensembl databases are built using MySQL and data input and analysis pipelines are written in Perl, normally utilising the eHive (49) workflow management system. While our database schema is subject to change, our Perl API is stable with changes deployed and announced in a controlled way. Specifically, we aim to support deprecated functionality for at least a year to provide ample time for those using our API in their pipelines to make the necessary updates.

Reducing sequencing costs have facilitated a rapid increase in the quantity of variant data available for a number of species, motivating us to regularly revise and optimize our analysis and storage methods. Key compute-intensive API functions, such as checking whether a variant overlaps another genomic feature, have been rewritten in C and can be optionally used through the Perl-XS interface. This brings considerable performance improvements when analysing large numbers of variants. We have also modified our API to use tabix (50), an efficient file access tool, to extract genotype data from Variant Call Format (51) files, removing the need to load large datasets into MySQL. Variant locations are stored in databases, enabling look up by names such as dbSNP refSNP identifier or ClinVar accession, followed by rapid extraction of genotype and allele frequency data from files.

To ensure our tools and data are compatible with other systems, we champion standards for data formatting and have adopted and contributed to the development of many standards. We drove the collaboration to develop the SO and use SO terms to describe both the type of change a variant represents and its consequence on overlapping genomic features (24). Consequences are annotated on the immutable Locus Reference Genomic (52) transcripts as well as the current Ensembl gene set. All variants are annotated using the HGVS (53) nomenclature, which has become the preferred way to describe variants in the clinical community. HGVS descriptions using Ensembl, RefSeq and LRG transcripts are provided where possible.

## Discussion

Genome browsers provide an integrated view of biological knowledge. This is essential to aid understanding basic questions about biological function, to provide data for evolutionary studies, and as a basis for genomics to have an impact on healthcare. Ensembl is one of a small number of projects providing variation data within a genome browser. The University of California, Santa Cruz Genome Browser provides a set of detailed and configurable views of genes, variants and other features but does not support as many species as Ensembl and does not currently provide REST API access. dbSNP provides the most detailed information available on the discovery of individual variants, but provides more limited browser capabilities and programmatic access.

## Future work

There are now a multitude of different algorithms available to predict the potential pathogenicity of human variants. We already provide 15 algorithms via VEP and later this year will add further predictions to our variant consequence views, alongside the existing SIFT and PolyPhen2 results. We will also extend the information we make available for interpreting variants outside coding regions. Making sense of large number of, often conflicting, results can be a challenge. We will employ simple colour-coding to provide a results overview and configurable tables to allow algorithm and score selection.

We will also enhance the protein annotations we provide and display variants in the context of protein structures. The number of phenotype and disease annotations in the public domain is increasing; between Ensembl release 56 and 93, the number of short variants with such annotations has risen from less than a thousand to more than 300 thousand. In this time, the proportion of variants with only a single annotation has dropped from 90% to 67% although a key problem is redundancy of information across different resources. We will continue to improve our existing methods to link related information, merge identical records relating to the same assertion and provide detailed provenance to enable filtering and extraction of preferred subsets of data.

Future development will also focus on integrating data at increasing scale. We anticipate large numbers of additional species will be sequenced for variant detection, while the number of sequenced individuals within many species continues to rise (54, http://gnomad.broadinstitute.org/, http://www.1000bullgenomes.com/). For human, this will improve the already extensive catalogue of rare variation across different global populations. Federation with other data distributors is the key to our plan for supporting this increase in the number of species studied and sample depth. Responsibility for the accessioning of variant data for all species but human is transferring from dbSNP to the EVA, providing opportunities for streamlining our processes and data storage. We already display genotype data direct from EVA for variants within the Ensembl system and will extend this functionality to provide Ensembl browser views of variant data held entirely within EVA.

We have played a role in the Global Alliance for Genomics and Health (55), a project whose aim is to accelerate progress in human health by developing harmonized approaches to enable effective and responsible sharing of genomic and clinical data. In particular, we are involved in defining standard models and data exchange formats for variant and variant annotation data. We will adopt relevant standards to be able to consume data from other key resources and to ensure we maximize the interoperability and usability of the data we provide in Ensembl.

Efficient data extraction methods and intuitive displays will be essential to derive full benefit from these increasing data types and volumes. We are currently engaged in a complete redesign of the Ensembl website and seek to implement simple views for the novice and detailed, configurable results selection and extraction tools to meet the specific needs of domain experts.

## Conclusion

Ensembl creates unique tools and visualisation for variation data and makes them freely available to the global scientific community. The data are comprehensively updated 4 times per year; each new release incorporates the current public knowledge for approximately 20 species and makes data available through a set of mature and stable interfaces. All data and software developed within the project are freely available. Archive web sites are maintained for at least 5 years to support replication of analyses. Our infrastructure is species agnostic and is used by other projects, such as Ensembl Genomes (56) and GRAMENE (57). We facilitate advances in genomic science by the provision of robust tools and comprehensive data sets to expedite analysis.

## Notes

**Database URL:** https://www.ensembl.org
